# TSH Receptor Signaling Abrogation by a Novel Small Molecule

**DOI:** 10.3389/fendo.2016.00130

**Published:** 2016-09-27

**Authors:** Rauf Latif, Ronald B. Realubit, Charles Karan, Mihaly Mezei, Terry F. Davies

**Affiliations:** ^1^Thyroid Research Unit, James J. Peters VA Medical Center, Icahn School of Medicine at Mount Sinai, New York, NY, USA; ^2^Sulzberger Columbia Genome Center, Columbia University, New York, NY, USA; ^3^Department of Pharmacological Sciences, Icahn School of Medicine at Mount Sinai, New York, NY, USA

**Keywords:** TSH receptor, small molecule, antagonist

## Abstract

Pathological activation of the thyroid-stimulating hormone receptor (TSHR) is caused by thyroid-stimulating antibodies in patients with Graves’ disease (GD) or by somatic and rare genomic mutations that enhance constitutive activation of the receptor influencing both G protein and non-G protein signaling. Potential selective small molecule antagonists represent novel therapeutic compounds for abrogation of such abnormal TSHR signaling. In this study, we describe the identification and *in vitro* characterization of a novel small molecule antagonist by high-throughput screening (HTS). The identification of the TSHR antagonist was performed using a transcription-based TSH-inhibition bioassay. TSHR-expressing CHO cells, which also expressed a luciferase-tagged CRE response element, were optimized using bovine TSH as the activator, in a 384 well plate format, which had a *Z* score of 0.3–0.6. Using this HTS assay, we screened a diverse library of ~80,000 compounds at a final concentration of 16.7 μM. The selection criteria for a positive hit were based on a mean signal threshold of ≥50% inhibition of control TSH stimulation. The screening resulted in 450 positive hits giving a hit ratio of 0.56%. A secondary confirmation screen against TSH and forskolin – a post receptor activator of adenylyl cyclase – confirmed one TSHR-specific candidate antagonist molecule (named VA-K-14). This lead molecule had an IC_50_ of 12.3 μM and a unique chemical structure. A parallel analysis for cell viability indicated that the lead inhibitor was non-cytotoxic at its effective concentrations. *In silico* docking studies performed using a TSHR transmembrane model showed the hydrophobic contact locations and the possible mode of inhibition of TSHR signaling. Furthermore, this molecule was capable of inhibiting TSHR stimulation by GD patient sera and monoclonal-stimulating TSHR antibodies. In conclusion, we report the identification of a novel small molecule TSHR inhibitor, which has the potential to be developed as a therapeutic antagonist for abrogation of TSHR signaling by TSHR autoantibodies in GD.

## Introduction

The TSH receptor (TSHR) is primarily expressed in the basolateral surface of thyroid follicular cells and induces thyroid cell growth, hormone synthesis, and hormone secretion and also happens to be a primary autoantigen in autoimmune thyroid disease; especially Graves’ disease (GD) (1–4). GD is one of the most common organ-specific autoimmune diseases with a prevalence of ~2% in the general population (5). It is an antibody and T cell-mediated disease where hyperstimulation of the thyroid gland leads to excess thyroid hormone production. The pathogenic effects of GD are driven, in part, by the interaction of stimulating antibodies to the TSHR, which bind to its large extracellular domain (ECD) (6). Such autoantibodies come in different varieties that can stimulate, block, or lead to apoptosis *via* induction of cellular stress (2, 7). In addition to its primary site on the thyroid cell, the TSHR is also expressed in a variety of extra thyroidal tissues where it is known to modulate target cell function, including fibroblasts and adipocytes and osteoclasts and osteoblasts (8–13). For example, there is evidence for a role of the TSHR in Graves’ orbitopathy and retro-orbital adipogenesis (13, 14) and as a negative regulator in bone remodeling (11). The presence of the TSHR in these and other extra thyroidal depots (10) makes it an important candidate receptor for several undefined roles secondary to the cascade of effects that may result from its chronic stimulation in GD.

In the last few years, small molecules have gained momentum as therapeutic options secondary to the development of large chemical libraries and robust high-throughput screening (HTS) assays (15). In addition to their low cost and ease to manufacture, they also have inherent chemical and biological advantages. These advantages include their ease in crossing plasma membrane barriers and their *in vivo* stability due to their resistance to proteolytic enzymes. Small molecule agonists against the TSHR have been reported by others (16, 17), as well as ourselves (18). However, to date, only a single TSHR antagonist has been reported, which was found following chemical modification of an agonist, but its potency is only in the micro molar range (19). There is now a need to improve the potency of such molecules to achieve a therapeutic IC_50_ in the nano molar range (10^−9^M).

All small molecules interacting with the TSHR appear to permeate the cell and dock with distinct polar and non-polar residues within the hydrophobic pockets created by the helices of the transmembrane (TM) domain and exert a stimulatory or inhibitory effect by altering the interaction and movement of these helices (20, 21), thus acting as novel pharmacophores. This report describes the identification and *in vitro* characterization of a small molecule antagonist to the TSHR selected by a chemical library screen using an in-house luciferase-based high-throughput inhibition assay.

## Materials and Methods

### Materials

Bovine TSH (1 IU/ml), human FSH (70 IU/ml), hCG (10 IU/vial), and forskolin (FSK) were purchased from Sigma-Aldrich (St Louis, MO, USA). The Bright-Glo™ luciferase substrate (Cat # E2610) was purchased from Promega Corporation, Madison, WI, USA. The cell culture medium, DMEM, and Ham’s F12 were purchased from Mediatech Inc., Manassas, VA, USA. Fetal bovine serum and fetal calf serum were purchased from Atlanta Biologicals, Flowery Branch, GA, USA. Additional amounts of lead compounds that were identified by screening were purchased from Enamine Inc., Cincinnati, OH, USA.

### Screening Libraries

Three libraries were used in the screening: (1) Lead-Optimized Compound library (LOC) made up of 9,690 molecules, (2) Enamine library made of 60,638 molecules, and (3) Analyticon library made up of 10,000 molecules. All three libraries were specifically designed by the Columbia University HTS facility (22, 23). A total of 80,328 molecules were screened as a single point, at a concentration of 16.7 μM. All potential hits were than analyzed by dose–response studies in triplicate.

### Cell Lines Used

(a) *CHO-HA-TSHR luciferase cells*: For HTS, we used cells generated by transfecting the pGL4.29 [luc2P/CRE/Hygro] construct into a highly selected stable line of CHO cells expressing the human TSH receptor with an hemagglutinin (HA) tag at the *N*-terminus (CHO-HA-TSHR cells) that has been previously described and was selected as a stable line with hygromycin (18). The cells were cultured in Ham’s F-12 medium with 10% fetal bovine serum (FBS) and 100 IU/ml of penicillin, 100 μg/ml streptomycin, and 50 μg/ml of hygromycin.
(b) *Murine Sertoli cell line TM4*: These FSH receptor expressing cells were obtained from ATCC (CRL-1715) and cultured in DMEM: F12 medium (cat # 30-2006) with 2.5% FBS and 5% horse serum (ATCC; cat #30-2040).
(c) *LH receptor-expressing cells*: The specificity against the LH/hCG receptor was tested using a stable line of rat LH/hCG receptor expressing HEK 293 cells that were kindly provided by Dr. K. M. J. Menon, University of Michigan, Ann Arbor, MI, USA. These cells were cultured in DMEM medium with 10% FBS and 100 IU/ml of penicillin and 100 μg/ml streptomycin.

### HTS Inhibition Assay

This assay was based on the same principle as described previously (18) for screening of agonist molecules. Briefly, 15,000 CHO-HATSHR Luci #1 cells [named TSHR-Glo cells (15)] were plated into white standard 384 wells at a volume of 30 μl in Ham F12 complete medium and incubated overnight at 37°C at >85% humidity. Library compounds were added at 16.7 μM to each well using a 384 nano-head (Perkin Elmer Inc.) and preincubated for 1 h at 37°C prior to stimulant addition. The compound added wells were then stimulated with 5 μl corresponding to 20 μU of bovine TSH for 4 h. To determine the luciferase activity in these cells at the end of incubation, the wells received 13 μl of the substrate Bright-Glo™. The luminescence was then measured using an EnVision multilabel reader (Perkin Elmer Inc.). In principle, activation of the TSHR by TSH results in G_sα_-adenylate cyclase coupling and an increase in intracellular cAMP, which results in the activation of CREB and its binding to the CRE element and subsequently induces the transcription of the *luciferase* gene and accumulation of the luciferase enzyme within the activated cells. Since the cells are preincubated with compounds that may inhibit the activation of Gsα-adenylate cyclase system, TSH activation of the receptor would be inhibited if the compound is a specific TSHR antagonist. However, the screen may result in false positives that inhibit activation of adenylate cyclase directly and thus inhibiting cAMP generation. Therefore, hits that are picked up in an initial screen must, then, be tested against FSK to rule out such false positives.

**Figure 1:**
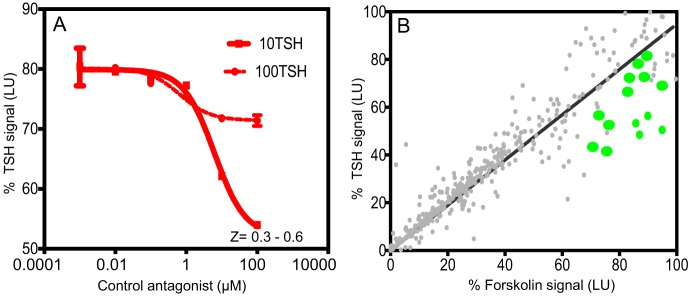
**Inhibition assay for detection of TSHR antagonists and overview of positive hits**. **(A)** The HTS luciferase assay that was developed for detection of antagonists against the TSHR was first tested for its TSH inhibitory activity using bovine TSH (10 and 100 μU) against a control antagonist. We screened a diverse chemical library of ~80,000 compounds for novel antagonists using this assay. **(B)** All positive hits were tested in parallel against TSH (*y*-axis) and forskolin (*x*-axis) as shown in this *x*–*y* plot. Molecules that showed >50% inhibition of TSH signal but 0 or <5% inhibition of forskolin signal were considered as potential inhibitors of the TSHR. Fourteen such randomly selected representative molecules are shown as green dots on this plot.

Throughout the screen, the signal to background ratio was linear and the mean CV was 5.4% and the Z′ factor was in the range of 0.3–0.6 based on the positive control Antag3 (19) (also kindly provided by Dr. M. Gershengorn, NIH, Bethesda, MD, USA) used in the plate. This exceeded the commonly accepted threshold for validation of high-throughput assays (24). When we challenged the cells with two different concentrations of bovine TSH (10 and 100 μU) (Figure 1A), we found that stimulation with 10 μU of TSH gave an inhibition of ~30–40% compared with less than 10% inhibition observed by stimulation with 100 μU of TSH. However, on optimization of the HTS, we found 20 μU TSH as the best stimulation because it gave similar inhibition in the HTS. We used an arbitrary fixed criteria for selecting molecules as positive hits if they showed ≥50% inhibition of TSH activity. Medium with <1% of DMSO was the negative control, whereas the control molecule with TSH and just TSH alone acted as positives in the assay for normalization of the signal and identification of positives hits.

### Confirmatory Assays

Dose–responses of the lead molecules were performed against TSH and FSK using a Tecan HP digital dispenser by following a similar protocol as described. All data points of the dose–response curves were fitted using Prism 5.0. A fluorescent viability assay (Cell Titer-Fluorviability assay, Promega Inc.) was also performed in the same experiment to assess toxicity of the molecules.

### Docking of Lead Molecule on the TSHR Transmembrane Domain

Docking of the lead molecules was performed on a homology model of the TSHR-TMD based on rhodopsin (PDB:1F88). This template was chosen because of the low root-mean-square deviation (RMSD) values between the backbone of the TM helices of the TSHR model and that of the rhodopsin X-ray crystal structure (25) and fits the experimental parameters that we have previously described (26). The initial homology model of rhodopsin was obtained from the Uniprot server.^1^ The conformations of the extracellular loops were constructed with the Monte Carlo method (27). The 3D geometries of the molecules in Tripos’mol2 format were generated with MarvinSketch.^2^ Docking was carried out using the docking, Autodock 4. The docking results were analyzed using DOCKRES and other supporting script tools (28).

### Serum Samples

Serum samples used in this study were unidentifiable stored samples originally collected with the full consent of patients.

### Statistical Analyses

All curve fitting and EC_50_ calculations were performed using GraphPad Prism version 5.02, and statistical differences for *P* values were calculated using one-tailed *t* test using Graph Pad In Stat software.

## Results

### Evaluation of the HTS Luciferase-Based Inhibition Assay for Screening and Identification of TSHR-Specific Inhibitors

**Figure 2:**
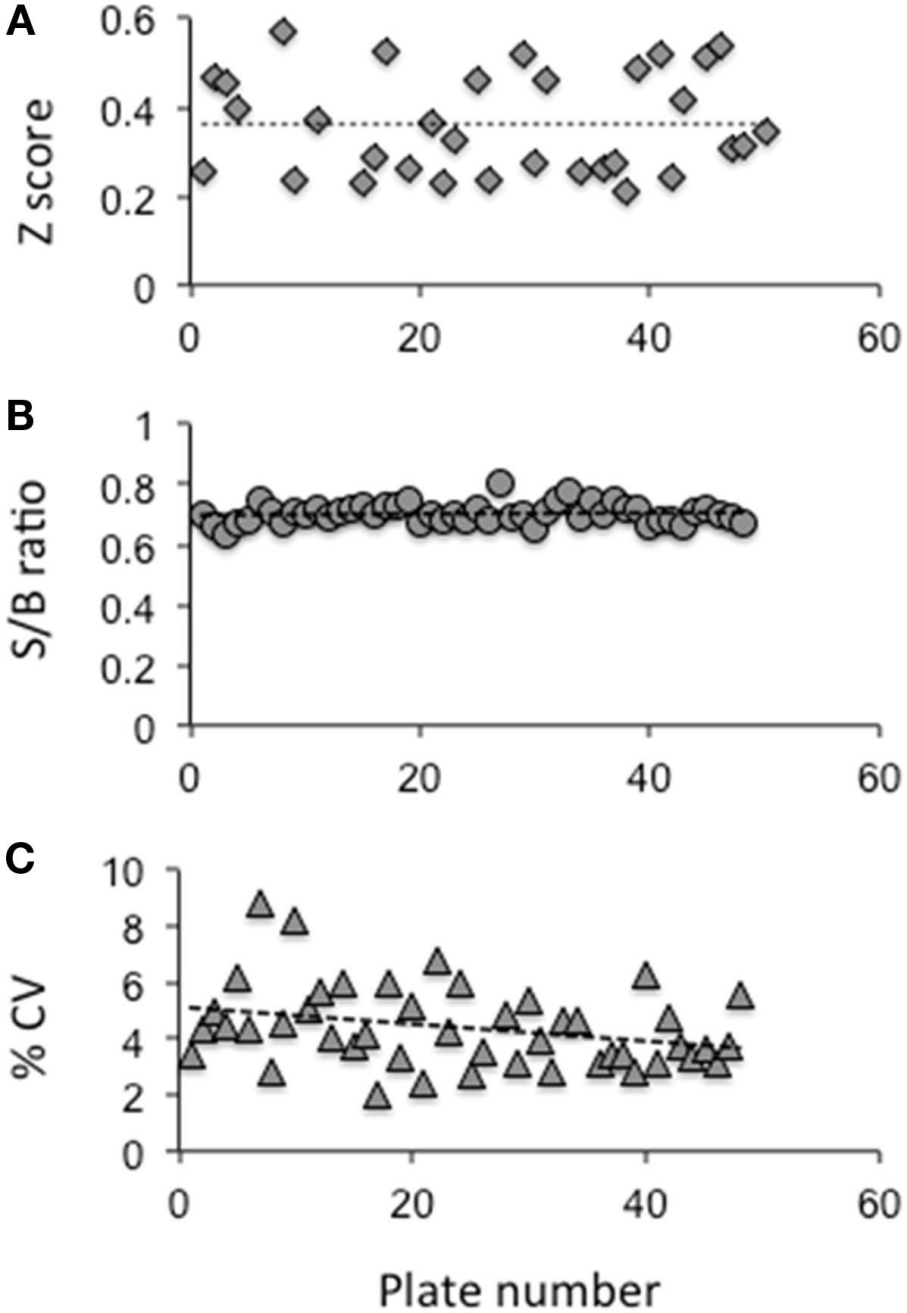
**Evaluation of the HTS assay**. **(A)** The *Z* factor was calculated using the positive control and basal control responses in each plate as per the formula described by Zhang et al (24) **(B)** The signal to background ratio was obtained using the total signal from the positive control well against those wells receiving medium plus DMSO. **(C)** The coefficient of variation (% CV) was calculated as the SD from the wells with the basal medium divided by the wells of the positive control. The data indicated that the HTS assay performed within the limits of a reliable screening.

Using this HTS inhibition assay, we screened a total of 80,328 molecules consisting of all three libraries as described earlier at a single concentration of 16.7 μM. We obtained 450 positives hits from this initial screen with a hit ratio of 0.56%. The performance of the assay throughout the screen is indicated by the *Z* score, signal to background ratio, and % CV plots (Figure 2). Further, to eliminate false positives and to obtain a secondary confirmation of the positives, we performed a secondary testing at 16.7 μM against 20 μU of TSH and 10 μM of FSK. Figure 1B is an *x*–*y* plot showing the results of such a screen where percent of TSH luciferase signal is indicated in the *y*-axis with percent of FSK signal in the *x*-axis. Molecules that showed 50% or greater inhibition of TSH and none or very little inhibition against FSK were generally regarded as potential inhibitory molecules specific to the TSHR and marked out for dose–response analysis. Fourteen such potential hits (marked by green circles) in Figure 1B are represented in the plot.

### Selection of a Specific TSHR Antagonist

**Figure 3:**
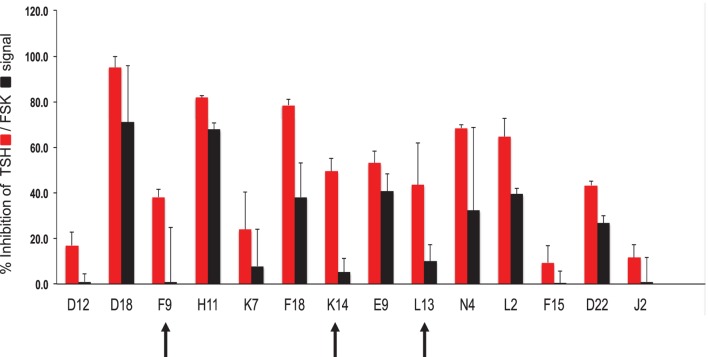
**Hit validation**. This bar graph illustrates the testing of the fourteen chosen compounds for inhibition of TSH and forskolin (FSK) signaling (mean ± SEM). The percent inhibition of the maximum TSH or forskolin signal is represented on the *y*-axis. Indicated by the arrows were three potentially specific candidates that showed minimal inhibition of forskolin (black bars) but significant inhibition of TSH (red bars). These three candidate molecules were then subjected to dose–response analyses.

Using our selection criteria, we identified 14 molecules as potential inhibitory molecules. These 14 molecules were further tested in triplicate at 16.7 μM against stimulation with 20 μU of TSH and 10 μM of FSK to confirm their specific inhibitory potency as indicated (Figure 3). Three molecules (marked by the arrows) appeared to have potential and were subjected to dose–response studies against TSH and FSK.

**Figure 4:**
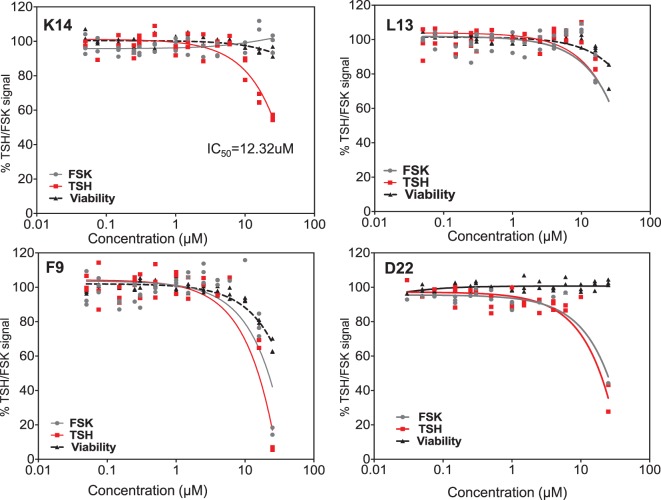
**Testing of selected lead molecules**. These four panels indicate the dose–responses of three likely (from Figures 3 and 1) and one control unlikely lead molecule against TSH (red) and FSK (gray). VA-K14 was the only molecule that effectively inhibited the TSH signal and had no inhibition for FSK compared with L13, F9, and D22. The toxicity of these molecules was tested by measuring viability (black) using the Cell titer Fluor assay within the same assay.

Dose–responses of each of the selected molecules (K14, L13 and F9) are represented in the different panels in Figure 4 along with one non-specific molecule (D22). The dose–response curves of the molecules strongly indicated that K14 had a 30–40% inhibition of TSH with negligible inhibition of FSK and low cytotoxicity compared with molecules L13, F9, or D22. Since K14 showed specific inhibition in the range examined, this was regarded as our most specific lead antagonist against the TSHR (now referred to as VA-K-14). VA-K-14 had an IC_50_ = 12.32 μM, and, although it was specific to the TSHR, it appeared to have a narrow inhibitory range.

### Specificity Analysis of VA-K-14

**Figure 5:**
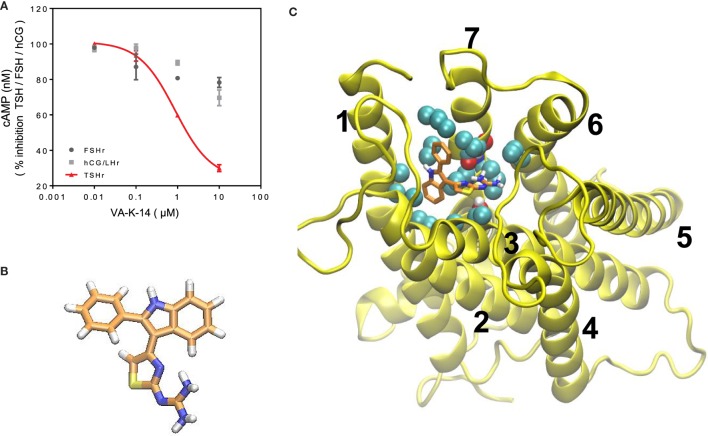
**(A)** Specificity and TSHR docking of the lead molecule **(A)** CHO-TSHr stable cells, HEK-LH/CGr stable cells, and FSHr- Sertoli cells (TM4) were stimulated with the maximum effective concentrations of their respective ligands as described in Section “Materials and Methods” for 1 h at 37°C in the presence of 1 mM IBMX and increasing concentrations of our lead antagonist VA-K-14. As indicated by the red line, VA-K-14 inhibited TSH by 75% at 10 μM in contrast to an inhibition of 10–15% with the hCG/LHr cells and FSHr cells. These data were average plots of two independent experiments performed in triplicate. **(B)** The structure of VA-K-14 is *N*-methyl-4-(2-phenyl-1H-indol-3-yl)-thiazole-2-amine with a molecular weight of 305.406 Da. **(C)** *In silico* docking performed on the homology model of the TSHR transmembrane domain (26) using Autodock 4 strongly suggested that VA-K-14 docks into the hydrophobic pocket of the TSHR–TMD, thus making contact with two residues in extracellular loop 1 (ECL1) (residues Asparagine 483 and tryptophan 488) and further contacts with Leucine 468 on TMH 2, Threonine 500 on TMH 3, and Valine 664 on TMH 7.

We, next, analyzed the specificity of VA-K-14 against other closely homologous glycoprotein receptors – the FSH receptor and LH/hCG receptor – using a cAMP femto HTRF bioassay (Cat # 62AM5PEB, Cisbio Inc.). For the LH receptor cells, we used HEK 293 cells transfected with the rat LH/hCG receptor, and, for the FSH receptor, we used a murine Sertoli cell line (TM4), which expresses the FSHR and responds to human FSH in a dose-dependent manner. Inhibition of intracellular cAMP generation was measured after stimulation of these cells with maximal responsive doses of their respective ligands (TSH, FSH, and hCG), after preincubation with VA-K-14 (0.01–100 μM). The TSHR-CHO cells were stimulated with 20 μU of bovine TSH, LH/hCC receptor cells with 1000 μU/ml of hCG, and Sertoli cells were stimulated with 700 μU/ml of human FSH, which had previously been titrated for optimum stimulation of cAMP under our experimental conditions (18). VA-K-14 showed more than 40% inhibition on the TSHR-expressing cells (Figure 5A). VA-K-14 showed a minor degree of inhibition (~10–15%) against the hCG/LH and FSH receptor-expressing cells, suggesting small molecules that are strong antagonists against the TSHR might have inhibitory effects against their homologous glycoprotein hormone receptors as seen previously (19).

### Define the Binding Sites by Molecular Docking

Inhibitory small molecule ligands are usually allosteric modulators of GPCRs (29). Figure 5B shows the molecular structure of VA-K-14 – which is *N*-methyl-4-(2-phenyl-1H-indol-3-yl)-thiazole-2-amine with a molecular weight of 305.406 Da. This molecule meets the Lipinski rule-of-five criteria (30) with an *x*log *P* of 4.34 and tPSA of 40. It is dissimilar in structure to the one published TSHR antagonist (19).

We examined the binding sites of VA-K-14 to the TM region of the TSHR by *in silico* docking, using a structure of the TSHR-TM region developed in our laboratory by homology modeling based on the rhodopsin crystal structure (26) and Monte Carlo simulations. By examining the top scoring docking poses generated by Autodock 4 and Autodock-Vina (all clustered at the same region of the extracellular hydrophobic pocket), we were able to deduce the putative contact residues within the TSHR-TM domain (Figure 5C). Docking analysis indicated that VA-K-14 is likely to make contact with residues Asn 483 (N483) and Trp 488 (W488) in ECL1 and Leu 468 (L468) on TMH1, Thr500 (T500) in TMH3, and Val 664 (V664) in TMH7 within the previously described hydrophobic pocket of the TMD (20, 21).

### Inhibition of TSHR Antibodies

**Figure 6:**
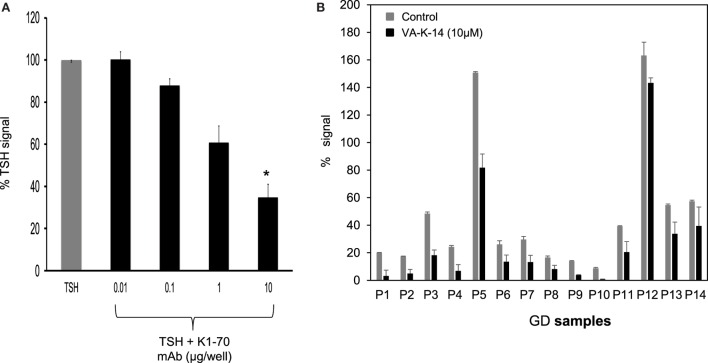
**Inhibition of GD sera by VA-K-14**. **(A)** The bar graph shows the inhibition of TSH signal (50 μU/ml) by a human blocking monoclonal antibody (K1-70). Increasing doses of antibody caused inhibition of TSH signal. Significant inhibition (*P* = 0.0116) was observed at 10 μg/ml of K1-70 monoclonal antibody. This suggested that the inhibition assay was capable of measuring TSHR-Ab inhibition of cAMP generation. **(B)** We tested a series of GD serum samples at 1:10 dilution for inhibition by VA-K-14. The cells were first preincubated with 10 μM of VA-K-14 or just medium and then challenged with diluted serum in triplicate wells. As indicated here, there was a varied degree of inhibition observed in the presence of 10 μM of VA-K-14 (black filled bars) compared with untreated serum (gray bars) with the luciferase assay. As seen here, P3 and P5 showed the most significant suppression of their stimulating responses in the presence of antagonist, but inhibition of P12 was poor.

We first tested our luciferase assay for inhibition of signal response in the presence of potent blocking TSHR antibody that binds to the ectodomain of the receptor. Figure 6A shows the inhibition of luciferase signal observed on stimulation of cells with 50 μU of TSH in the absence of antibody (gray bar) and in the presence of increasing doses of a monoclonal human TSHR blocking antibody (K1-70) (31) (kindly provided by Dr Bernard Rees Smith, RSR Ltd., Cardiff, Wales). Nearly 40% inhibition of stimulation was observed at 10 μg of the K1-70 blocking antibody.

In order to assess if our lead antagonist VA-K-14 was capable of inhibiting stimulating TSHR antibodies in GD patient sera, we tested a panel of 14 Graves’ sera (diluted 1:10) in the absence and presence of 10 μM of VA-K-14. We observed variable degrees of inhibition of stimulation although all the sera were inhibited to some degree (Figure 6B). VAK-14 was also effective in inhibiting a widely used human monoclonal-stimulating antibody [M22, also kindly provided by Dr Bernard Rees Smith, RSR Ltd., Cardiff, Wales (32)] and a hamster-derived stimulating monoclonal antibody (MS-1) (33) (Table 1).

**Table 1:** **Dose-dependent inhibition of M22 and MS1 by molecule VA-K-14**.

Dose of VA-K-14 (µM)	M22 (% change)	MS1 (% change)
0	100	100
0.1	99.93 ± 2.36	95.64 ± 4.56
1	83.63 ± 3.38	90.73 ± 0.91
10	68.36 ± 0.49	64.47 ± 1.00

*Note: the dose of M22 used for stimulation was 1.5 µg final and that of MS-1 was 15 µg, final. The assay was carried out with 15,000 cells/well in 348 well plate using CHOTSHR luciferase cells*.

### Combining Two Structurally Dissimilar Antagonists

**Figure 7:**
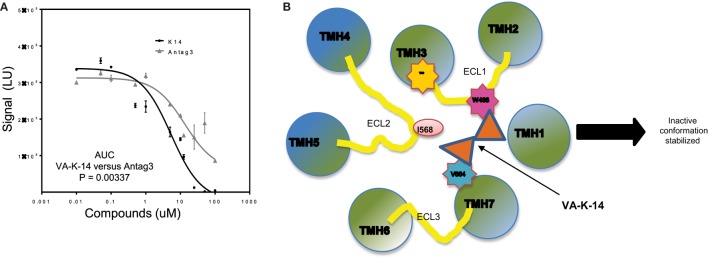
**Mechanistic action of VA-K-14**. **(A)** Indicated in this dose–response graph is a comparison of our lead molecule VA-K-14 and the reported Antag3 in order to compare their inhibition of TSH signaling. Although the two molecules are structurally different, they clearly have the potential to inhibit TSH action in a similar manner. However, the area under the curve calculation of VA-K-14 versus Antag3 indicated VA-K-14 to be significantly different in its inhibitory characteristics of the TSH response in this assay. **(B)** This diagram indicates the possible mechanism for the inhibitory effect of VA-K-14 on binding to the hydrophobic pocket in the TMD of the TSHR. The molecule contacts tryptophan 488 (W488) in ECL1 (pink) and valine 664 (V664) on helix 7 (blue). W488 is a naturally occurring inactivating mutation where V664 is a critical partner with Isoleucine 568 in ELC2 in stabilization of the receptor conformation. Thus, it would seem that this charge interaction network formed by VA-K-14 in the hydrophobic pocket must play a role in stabilizing an inactive conformation of the receptor transmembrane and, thus, leading to dampening of the signal.

When we compared VA-K-14 with the control antagonist (Antag 3), which is structurally dissimilar and makes contact with disparate residues in the TMD region, we found similar degrees of inhibition at concentrations between 1 and 100 μM, but comparison of the area under the curves (AUC) showed that the two molecules were significantly (*P* = 0.003) different in their degree of inhibition (Figure 7A). This significant difference in their dose–response relationships also most likely indicated their binding to different residues in the TSHR-TM domain. Since VA-K-14 and Antag 3 have different binding sites within the hydrophobic pocket of the receptor, we examined the effect on inhibition of stimulation by combining the two. This analysis clearly indicated that combining two antagonists did not enhance the degree of inhibition (data not shown), suggesting that the complex dynamic molecular interactions that the small molecules make with the receptor allosteric site has limits to its distortion potential.

### Mechanism of VA-K-14 Action

Docking analysis indicated that VA-K-14 makes contact with tryptophan 488 (W488) on ECL1 and a second contact with Valine 664 (V664) in TMH 7 by launching itself in the hydrophobic pocket formed by helices of the TSHR TMD (Figure 7B). It is known that W488 is an important residue of a naturally occurring inactivating mutation (34) and V664 of helix 7 is a critical partner with Isoleucine 568 (35) on ECL2 which helps in stabilization of the receptor conformation.

## Discussion

Hyperthyroid GD is currently treated with antithyroid drugs, radioactive iodine, or surgery (36). However, these modes of treatment though effective are not without complications and antithyroid drug treatment is commonly followed by relapses (37, 38). Antithyroid drugs can also cause severe or even life-threatening complications. Nevertheless, there have been few attempts to develop newer drugs that would be more effective. Small molecule antagonists that can inhibit signaling of the TSHR do, however, have serious potential as novel therapeutic options. We have previously reported the development of two lead small molecule agonists against the TSH receptor (18). In this study, we describe the *in vitro* characterization of a novel TSHR antagonist identified by high-throughput screening using a transcriptional-based luciferase inhibition assay.

Though the TSHR is promiscuous in engaging several G proteins (39) and β-arrestin 1 and 2 (40–42), it is known that the predominant signal that comes from the receptor is *via* engagement of the Gs subclass of G protein, which leads to the generation of second messenger cAMP. We have previously exploited this major signaling pathway to develop a sensitive HTS assay for TSHR agonist detection, known as the TSHR-Glo assay (15). We extended the stimulation assay by first pre-incubating the cells with the library compounds and, then, stimulating them with an optimized dose of bovine TSH for 4 h before reading the luciferase signal. Though the inhibition HTS assay was not robust in its performance as just judged by *Z* factor score, it was effective enough to detect 30–40% inhibition of low dose TSH stimulation and maintained a fairly consistent *Z* score (Figures 1A and 2). However, on screening a diverse library of compounds of 80,328 molecules using this HTS assay, we identified only one lead molecule (VA-K-14) that was specific to the TSHR and failed to show any inhibition of FSK – a post receptor activator of cAMP – even in dose–response analysis (Figure 4).

Since the TM domain of major GPCR’s, especially the glycoprotein hormone receptor family, such as the FSH and hCG/LH receptors, is quiet homologous (43) in their sequence, it was important to examine receptor specificity of the lead small molecule. Using cells that express the FSH and LH receptor cells, we examined the specificity of our lead molecule VA-K-14. Though a potent inhibitor of TSHR, it also showed minor inhibition of cAMP generation against FSH and LH receptor bearing cells at the highest effective concentrations as seen with the previously reported antagonist (Antag 3) (19). The homologous nature of the TM domain of these receptors may cause such reactivity to be inevitable, and a potent antagonist, which was reported previously to be in the nanomolar range (44), was also found to lack all specificity against the TSHR and, thus, could not be developed further. But, it is known that functional ability of the allosteric modulators against the TSHR are defined by the contact residues within the TM domain (20), and the pharmacophore property of small molecules can be altered by structural alteration of their scaffold as evidenced by the development of Antag 3 (19).

To examine the contact sites of our lead molecule (VA-K-14), we docked the molecule with a well verified TM domain structural model that we previously developed on the rhodopsin template (26). From the docking studies, it was clear that VA-K-14 docked in the hydrophobic pocket of the TSHR-TM domain making polar and non-polar contacts with residues asparagine 483 (N483) and tryptophan 488 (W488) on the extracellular loop 1 (ECL1) and leucine 468 (I468) on TM 2, threonine 500 (T500) on TM 3, and valine 664 (V664) in TM 7. The contact residues of this molecule in the hydrophobic pocket are different from those of our lead agonists (18) and were also different from Antag 3.

It is known that GPCR activation could involve the movement of helices, especially TM3 and TM6 (45–47), and modeling studies with mutational analyses have clearly outlined several residues within the hydrophobic pocket defined by the various helices of the TMD (20, 21, 48). Hence, the molecular property of allosteric small molecules will reside in the mosaic of interactions that such a molecule makes within the pocket, thus stabilizing an active or inactive state of the TSHR. It is known that residue W488 in ECL1 is a naturally occurring inactivating mutation (34) and V664 on TMH7 is a critical partner with I568 in ECL in stabilizing a native receptor conformation (35). Thus, from the docking data, we can hypothesize that VA-K-14, by interacting with these two residues, may stabilize an inactive form of the TSHR, leading to inhibition of TSH signaling. Furthermore, a recent study (49) using computation analysis and chemical crosslinking followed by mass spectrometry has also shown that rearrangement of the ECD/ECL1 is critical for TSHR activation. Thus, any allosteric molecule that could thwart such a rearrangement (50) would be a potential negative allosteric modulator (NAM).

In conclusion, we identified and characterized, *in vitro*, a new antagonist of the TSHR, which is a potential candidate for further development to provide a therapeutic option for controlling the action of auto antibodies to the TSHR in hyperthyroid GD and its related manifestations.

## Author Contributions

RL: assay development and majority of the experiments and wrote manuscript. RR: did the screening of molecules. CK: coordinated the screening and data analysis. MM: did the docking work. TD: coordinate the project, data analysis and writing of manuscript.

## Conflict of Interest Statement

The authors declare that the research was conducted in the absence of any commercial or financial relationships that could be construed as a potential conflict of interest.

## Footnotes

^1^http://www.uniprot.org

^2^http://www.chemaxon.com

## Abbreviations

BD, Brownian dynamics; FSH, follicle-stimulating hormone; FSK, forskolin; HTS, high-throughput screening; LH, luteinizing hormone; MD, molecular dynamics; SML, small molecule ligands; TMH, transmembrane helix; TSHR, TSH receptor.
